# Magnetic ion channel activation of TREK1 in human mesenchymal stem cells using nanoparticles promotes osteogenesis in surrounding cells

**DOI:** 10.1177/2041731418808695

**Published:** 2018-10-30

**Authors:** James R Henstock, Michael Rotherham, Alicia J El Haj

**Affiliations:** 1 Institute of Ageing and Chronic Disease, University of Liverpool, Liverpool, UK; 2 Institute of Science and Technology in Medicine, Guy Hilton Research Centre, Keele University, Staffordshire, UK

**Keywords:** Magnetic nanoparticles, tissue engineering, mesenchymal stem cell, stretch-activated ion channel, paracrine

## Abstract

Magnetic ion channel activation technology uses superparamagnetic nanoparticles conjugated with targeting antibodies to apply mechanical force directly to stretch-activated ion channels on the cell surface, stimulating mechanotransduction and downstream processes. This technique has been reported to promote differentiation towards musculoskeletal cell types and enhance mineralisation. Previous studies have shown how mesenchymal stem cells injected into a pre-mineralised environment such as a foetal chick epiphysis, results in large-scale osteogenesis at the target site. However, the relative contributions of stem cells and surrounding host tissue has not been resolved, that is, are the mesenchymal stem cells solely responsible for the observed mineralisation or do mechanically stimulated mesenchymal stem cells also promote a host-tissue mineralisation response? To address this, we established a novel two-dimensional co-culture assay, which indicated that magnetic ion channel activation stimulation of human mesenchymal stem cells does not significantly promote migration but does enhance collagen deposition and mineralisation in the surrounding cells. We conclude that one of the important functions of injected human mesenchymal stem cells is to release biological factors (e.g., cytokines and microvesicles) which guide the surrounding tissue response, and that remote control of this signalling process using magnetic ion channel activation technology may be a useful way to both drive and regulate tissue regeneration and healing.

## Introduction

**Figure 1.**
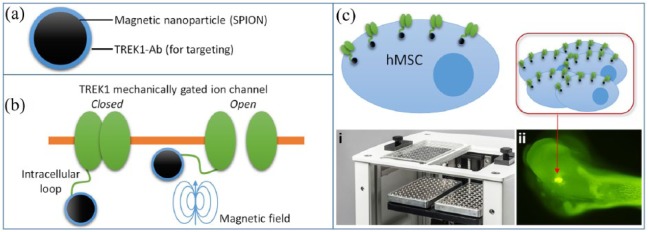
MICA activation of the TREk1 stretch-activated ion channel. (a) Superparamagnetic ion oxide nanoparticles (SPIONS) were surface functionalised with antibodies specific to the mechanosensitive intracellular loop region of the TREK1 ion channel. (b) Attachment of the nanoparticle to the ion channel allows the ion channel to be activated (opened) using an external magnetic field. (c) Tagging TREK1 in hMSCs allows remote control of mechanotransduction using magnets, such as the (i) MICA bioreactor moving magnetic array used in this investigation, and (ii) remote control of injected hMSCs as reported by Henstock et al.^4^

Magnetic ion channel activation (MICA) technology enables a level of remote control over the molecular functions of nanoparticle-tagged cells using magnets acting over a distance, that is, from outside the body.^1,2^ The MICA principle involves surface functionalising superparamagnetic iron oxide nanoparticles (SPIONs) with a biomolecule – commonly either an antibody or ligand.^3^ A moving external magnetic field then applies a dynamic force (torque) to the nanoparticle which delivers mechanical forces to the target, resulting in mechanotransduction or activation of downstream signalling (Figure 1). We have previously demonstrated that ion channels,^4,5^ integrins,^4–7^ and Wnt receptors^8^ can be activated using this method, allowing researchers external, electronic control over complex biological pathways and downstream stem-cell differentiation.

The TREK1 mechanosensitive ion channel can be remotely controlled using magnetic nanoparticles conjugated with an anti-TREK1 antibody, and that this acts as a powerful stimulus for driving bone repair.^2,4^ TREK1 is a two-pore-domain potassium channel expressed in multiple tissues.^6^ The mechanically gated TREK1 ion channel can be remotely activated by attaching conjugated nanoparticles to the intracellular loop region and applying an oscillating magnetic field, resulting in observable changes in whole-cell electrophysiology.^5^ Directing mechanotransduction via TREK1 has been shown to result in the osteogenic differentiation of mesenchymal stem cells (MSCs) and increased expression of both osteogenic genes (collagen I, osteopontin and CBFA1) and chondrogenic genes (SOX9 and collagen II).^5^ Developing the sophistication of this nanoparticle-based mechanotransduction technique using in vitro culture^5^ through to three-dimensional (3D) cell culture, organotypic ex vivo^4^ and in vivo^2,9^ models, we have demonstrated how mechano-stimulation of human mesenchymal stem cells (hMSCs) using magnetic nanoparticles results in differentiation towards the bone and cartilage lineage.^1,3^

Using a chick foetal femur model of endochondral ossification,^10^ we have previously reported the effects of injecting a population of hMSCs which had been tagged with TREK1-targeting nanoparticles into the cartilaginous epiphysis of an organotypically cultured foetal femur.^4^ After 14 days in culture, a large amount of de novo bone formation was observed throughout the epiphysis, particularly in the region immediately below the outer superficial layer of the tissue. In the magnet-stimulated femurs injected with MSCs tagged with TREK1 nanoparticles, an average of 31% more mineralisation was formed compared to controls. This substantial effect was generated from just a small number (10^3^) of injected cells, posing questions about the underlying biological mechanism that was being triggered. We developed two hypotheses: (1) the nanoparticle-tagged stem cells were migrating to the sub-surface of the epiphysis and directly producing bone, or (2) the bone formation was created by native chick cells in response to unknown factors secreted by the mechanically activated human stem cells. Both theories have generated some support in the literature, with some evidence of magnet-guided migration in nanoparticle-labelled rat bone marrow MSCs^11^ and emerging evidence the mechanotransduction results in the release of paracrine factors from MSCs that drive bone formation.^12^

Deciphering this mechanism in a complex, 3D, organotypic foetal tissue proved to be extremely technically challenging, so we simplified our methodology to investigate these two hypotheses under more controlled in vitro conditions. In this article, we report our results from (1) using a transwell migration assay to determine the effects of magnetic nanoparticles on hMSC motility and mobilisation and (2) a novel two-dimensional (2D) co-culture method for determining the paracrine effects of mechanically stimulated hMSCs on surrounding foetal chick epiphyseal cells (CECs).

The aims of this investigation are to shed further light on the mechanisms behind MSC responses to mechanoactivation and the subsequent effects on surrounding tissue homeostasis and repair. The overall goals are to examine how MICA nanoparticle technology can be used to remotely control the tissue regeneration process. Magnetic nanoparticles have existing regulatory approval, and their long-term safety has been demonstrated.^13,14^ The efficacy and mechanism of action for MICA technology is therefore of considerable interest for translational tissue engineering and regenerative medicine.

## Methodology

### Human MSC culture

Human MSCs were obtained from a bone marrow aspirate (Lonza, USA) and cultured to passage three in basal Dulbecco’s modified Eagle’s medium (DMEM) containing 10% foetal calf serum (FCS) and 1% penicillin–streptomycin. A single donor was used (24 years old healthy male).

### Magnetic nanoparticle labelling

Nanomag superparamagnetic nanoparticles of 1 mg (carboxyl-coated, 300 nm in diameter; Micromod, Rostock, Germany, http://www.micromod.de) were surface activated by washing in sterile 1-ethyl-3-(3-dimethylaminopropyl)-carbodiimide hydrochloride and *N*-hydroxysuccinimide in 0.5 M (2-(N-morpholino)ethanesulfonic acid) MES buffer, adjusted to pH 6.3 with Na_2_CO_3_ for 1 h at room temperature, recovered by magnetic separation, and washed in 0.1 M MES buffer. Nanoparticles (1 mg) were then conjugated to either 10 μg of RGD-tripeptide or 10 μg of TREK1-Ab (Alomone Labs, Jerusalem, Israel, http://www.alomone.com) by mixing together in 1 ml of 0.1 M MES buffer for 3 h. Attachment of the (arginylglycylaspartic acid) RGD-coated nanoparticles to their targets in the MSCs was achieved by culturing the hMSCs in suspension in serum-free media for 3 h followed by incubation with 125 μg of particles per 10^6^ cells with intermittent agitation. The cells were centrifuged, washed and immediately used in experiments. Because the TREK1 antibody epitope is intracellular, these particles were first coated in 40 ng of *N*-(1-(2,3-dioleoyloxy)propyl)-*N, N*, –trimethylammonium methyl-sulphate to aid nanoparticle uptake.

### Magnetic force bioreactor

Magnetically stimulated groups were placed in an incubator (37°C, 5% CO_2_) above a custom-built vertical oscillating magnetic force bioreactor (MICA Biosystems, West Midlands, U.K., http://micabiosystems.com), thus maintaining otherwise standard culture conditions. Nonstimulated control groups were kept in identical conditions (without magnetic field). Magnetically stimulated groups were exposed to a maximum 25-mT magnetic field from an array of permanent magnets (NdFeB) situated beneath the culture plates at a frequency of 1 Hz. Magnetic stimulation was performed in daily 1-h sessions as described below.

### Magnetic field mapping

The magnetic field strengths, polarities and gradients produced by the permanent magnetic arrays were mapped and modelled at a resolution of 0.2 × 0.2 mm using a Magscan 300 instrument (Redcliffe Magtronics, Dartford, UK). The field maps and polarities at varying distances were determined by altering the distance between the magnetic array’s and the detector up to a maximum distance of 84 mm from the array. This allowed estimations of the magnetic field gradients that were applied to the magnetic nanoparticle to be calculated.

### Transwell migration assay

To determine if SPION-labelled hMSCs migrate towards the magnetic gradient, hMSCs were labelled with the live cell tracker dye PKH26, using the manufacturers standard protocol (Phanos Technologies via Sigma Aldrich). PKH26-labelled cells (10^3^) were placed in upper chamber of a 6.5-mm FluoroBlok transwell (24-well plate) with 8 µm pores. The plates were then exposed to the magnetic force bioreactor for 1 h per day in line with our standard MICA protocols. Migration across the FluoroBlok membrane towards the oscillating magnet was quantified by directly quantifying fluorescence on the underside of the membrane after 96 h. The following negative controls were used: hMSCs alone, hMSCs incubated with blank (unconjugated) nanoparticles and with 30% FCS in the lower chamber acting as a positive control for migration.

### Chick foetal femur isolation and cell culture

Intact femurs were removed from freshly killed Dekalb white chick foetuses after 11 days of gestation and carefully cleaned of all muscle tissue by rolling on sterile tissue. Isolatedemurs measured approximately 7 mm at isolation. The epiphyses were removed from the femurs and incubated overnight in a 1 mg/ml solution of collagenase and centrifuged at 400 g to recover the epiphyseal chondrocytes, which were then allowed to attach and proliferate on tissue culture plastic (T-flasks) for 48 h in DMEM containing 10% FCS. The cells were cultured at 37°C and at 5% CO_2_ in a humidified incubator, with culture medium being completely replaced every 24 h.

### Human MSC chick co-culture assay

To determine generally enhanced migration and obtain evidence for any paracrine effects of nanoparticle mechano-activated cells on surrounding CECs, we established a localised co-culture assay. In this experiment, a 10 μl droplet containing 10^3^ PKH26-labeleld hMSCs was placed into the exact centre of a 6-well plate. These were allowed to adhere for 3 h, at which point 10^6^ CECs were seeded on top of the hMSCs in 2 ml media. A semi-osteogenic media was used as in the original organotypic culture experiment previously reported:^4^ DMEM containing 10% FCS, 1% penicillin–streptomycin and 150 μg⋅ml^−1^ ascorbic acid, 2 mM sodium β-glycerophosphate, and 10^−8^ M dexamethasone (all from Sigma Aldrich, Cambridge, U.K). The co-cultures were maintained for 28 days with media changes every 48 h and daily exposure to the standard MICA protocol described above (1 h per day). Controls of the hMSC droplet alone and the chick-derived epiphyseal cells alone were used. After 28 days, the spread of hMSCs from the initially seeding dot was quantified by area scan on a fluorescence plate reader. Alkaline phosphatase activity in the media was also quantified (protocol described below) and the plates were sequentially stained for calcium and total collagen, both of which were subsequently quantified (protocol described below).

### Alkaline phosphatase activity

Alkaline phosphatase activity in the culture medium was measured by taking a 50-μl sample of the medium and quantifying the dephosphorylation of *p*-nitrophenyl phosphate, a phosphatase substrate that turns yellow (*λ*_max_ = 405 nm) when dephosphorylated by alkaline phosphatase after 10 min of incubation at room temperature. Readings were obtained immediately and then at 5-min intervals to establish that data were acquired within the linear range of the assay. Results are reported following 10 min of incubation at room temperature.

### Calcium quantification

Media was removed from the plates and these were gently washed with phosphate-buffered saline (PBS). Calcium deposits on the plate were stained with a 1% solution of Alizarin red, which was then washed with water until residual unbound dye was removed. The plates were then imaged using a plate scanner. Quantification of total calcium in each well was by immersing the alizarin-stained samples in 5% cetylpyridinium chloride (Sigma Aldrich) solution for 2 h, yielding a purple destain solution containing the solubilised cetylpyridinium–alizarin complex, which was quantified in a spectrophotometer at 562 nm.

### Collagen quantification

Total collagen was quantified using the collagen stain, sirius red (Direct red 80, Sigma Aldrich), an anionic dye with sulphonic acid side chain groups which interact with the side chain groups of the basic amino acids present in collagen. Specific affinity of the dye for collagen under the assay conditions is due to the elongated dye molecules becoming aligned parallel to the long, rigid structure of native collagens that have intact triple helix organisation. A small quantity of (1 ml) 1% w/v sirius red S in dH_2_0 was added to each well and incubated at room temperature for 20 min, after which the unbound dye was removed and the wells were gently washed with water. The plates were imaged using the area scan function of the plate reader at absorbance 540 nm to determine where in the plate the collagen was being deposited. The collagen-dye complex was the dissociated in 1 ml 0.5 M NaOH, and the absorbance of the solution at 540 nm was measured in a spectrophotometer. The assay was calibrated against a standard curve of 12.5–100 μg/ml rat tail collagen (type-I collagen from rat tail, Sigma, UK).

## Results

**Figure 2.**
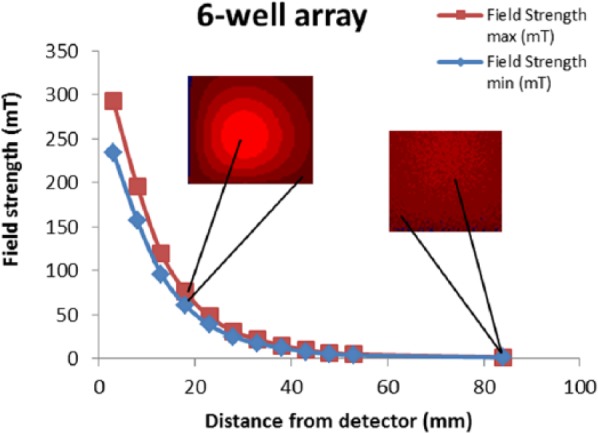
Magnetic field gradients. Graphical representation of the magnetic field gradients of the six-well permanent array used in this investigation. When the array was positioned towards the detector/culture, this resulted in a peak magnetic field range of 240–400 mT. Away from the sample, at a distance of 84 mm, the magnetic field strength was reduced to 1–2 mT. The images in red (inset) show representations of the field pattern experienced at the well base at each distance from the array.

**Figure 3.**
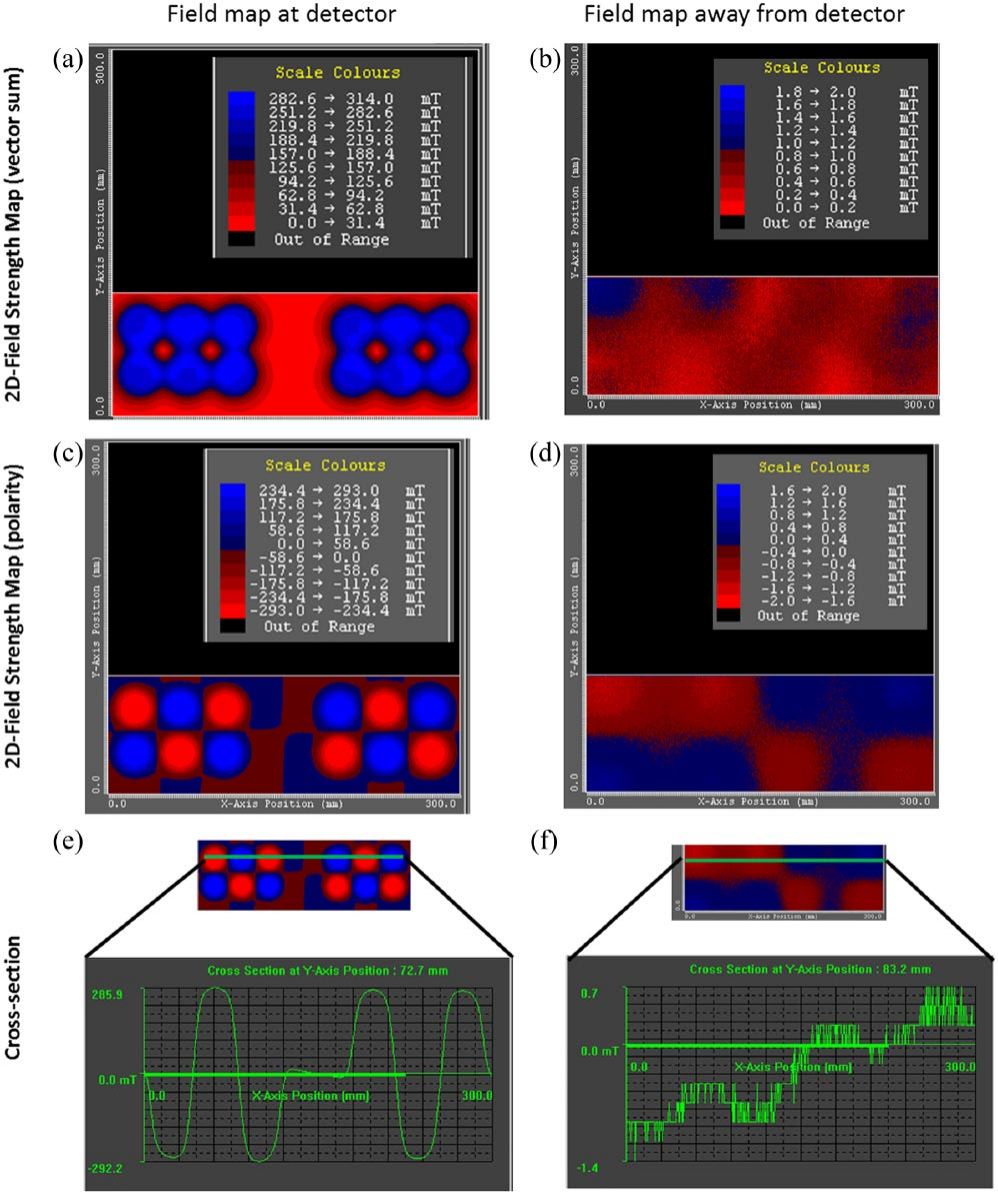
Six-well array magnetic field mapping. 2D model representations of magnetic field strength and polarity of six-well permanent magnet arrays. When the magnetic array was closest to the samples (approximately 18 mm), the peak magnetic field strength was ±75 mT (a), the peak field strength and field polarity is shown in (c). A representative cross-section of the magnetic fields and polarity is shown in (e). When the magnetic array was the furthest (measurable) distance from the samples (approximately 84 mm), the magnetic field strength was reduced to ±1.4 mT (b), the field strength and field polarity is shown in (d). A representative cross-section of the magnetic fields and polarity is shown in (f).

The magnetic field strengths of the arrays were measured across a range of distances in order to determine the applied magnetic field gradients experienced in vitro during the migration and mechanotransduction assays (Figure 2). The arrays displayed magnetic field strengths that peaked at approximately 300 mT when positioned close to the detector/well-plate base, and the magnetic field profiles appeared to be regular and well defined as shown by cross-sections of the magnetic field arrays (Figure 3). The magnetic field strength decreased exponentially as a function of distance from the detector with minimum field strengths of approximately 1 mT (at a distance of 84 mm), at this distance the magnetic field profile became irregular.

**Figure 4.**
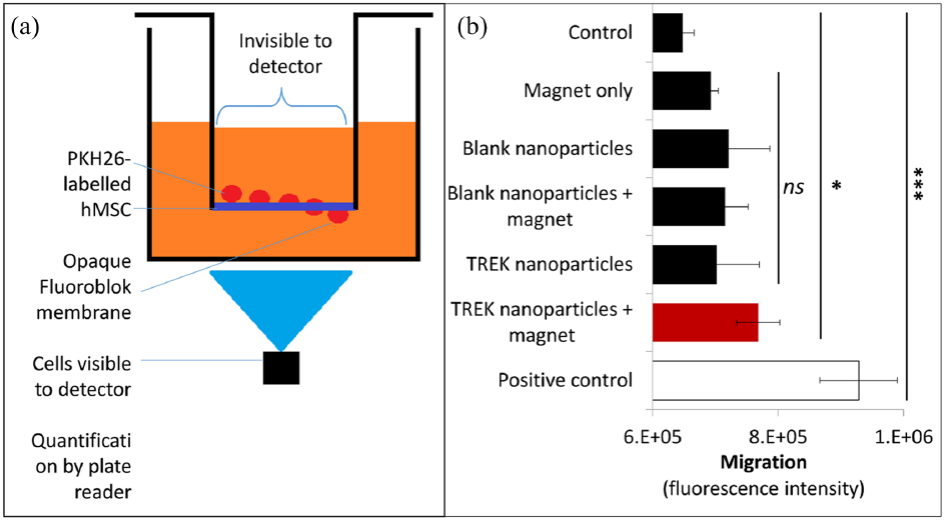
Transwell migration of hMSCs tagged with magnetic nanoparticles. PKH26-labelled mesenchymal stem cells (hMSCs) (10^3^ cells) were placed in the upper chamber of FluoroBlok transwell and migration across the membrane was quantified after 96 h. The relative migration of hMSCs labelled with either unconjugated (blank) or TREK1-targeting nanoparticles was compared to unlabelled cells (control) and unlabelled cells exposed to the magnet. High concentration foetal calf serum (30% FCS) served as the positive control. All groups exposed to the nanoparticles or the magnetic array showed a slight (non-significant) increase in migration over the control, and the TREK1-stimulated group (highlighted red) showed significantly increased migration (*p* = 0.02) over the control. Bars represent the experimental mean, and error bars show standard deviation, *n* = 6. *p < 0.05 and ***p < 0.001.

Our initial experiment used FluoroBlok transwell inserts to determine the migration of hMSCs tagged with TREK1-Ab conjugated nanoparticles towards the magnet (Figure 4), with the results reflecting the number of MSC which had migrated across the membrane towards the stimulus (either magnet or serum in the positive control). TREK1-tagged MSCs were found to migrate more towards the magnet (*p* = 0.02) than the MSC control group (which had neither nanoparticles nor magnet). However, all experimental groups (hMSCs tagged with unconjugated nanoparticles, untagged cells exposed to a magnetic field or TREK1-labelled cells without a magnet) all showed slightly increased migration. Although the TREK1-tagged hMSCs did migrate more than the control, the 19% increase was smaller than the 45% increased chemotactically driven migration towards FCS in the positive control group.

**Figure 5.**
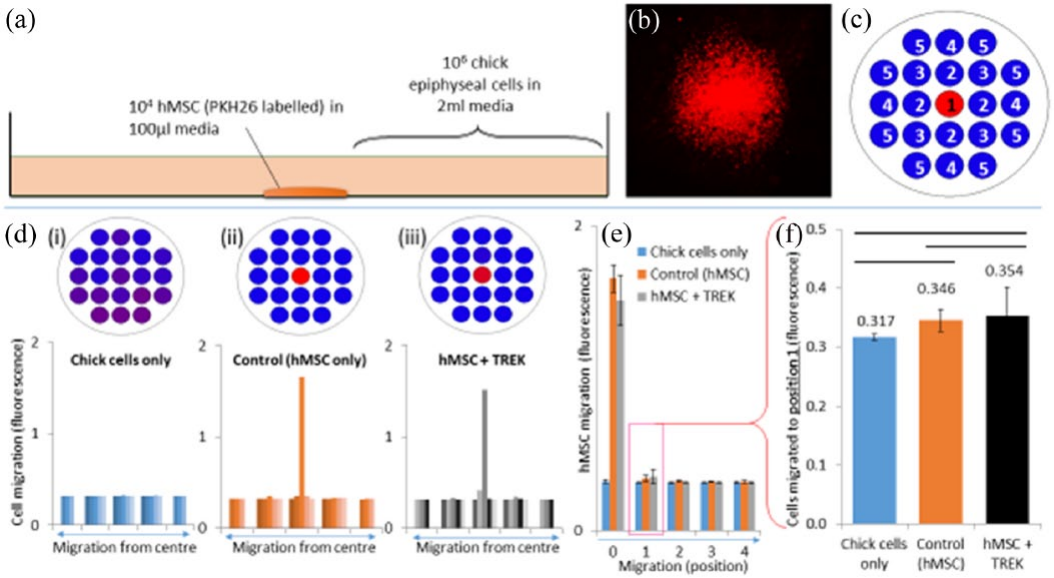
Migration of hMSCs into a co-culture of chick epiphyseal chondrocytes was not significantly enhanced by mechanotransduction. (a) PKH26-labelled hMSCs (10^3^) were placed in the centre of a well in a six-well plate and 10^6^ chick epiphyseal cells were seeded on top. The outgrowth of the PKH26-labelled hMSCs (b) from the central seeding dot was quantified after 28 days using the area scan function (c) of a fluorescence plate reader. (d) No significant migration was observed in either the control or TREK1 nanoparticle-tagged hMSCs. (e) Statistical analysis showed that there was no significant migration across the plate, and that the hMSCs remained in the same place for the duration of the experiment (28 days). (f) Error bars show standard deviation, *n* = 6.

Our next experiment sought to determine if the TREK1 nanoparticles caused enhanced migration perpendicular to the magnetic field. Fluorescently labelled (PKH26) hMSCs (10^3^) were placed in a drop in the centre of a six-well plate and allowed to adhere, after which 10^6^ CECs (derived from foetal femurs isolated at e11) were seeded across the entire well (Figure 5). The area scan function of a plate reader was used to detect the presence of fluorescent cells at measurement positions radially from the seeding position. After 28 days, we observed that there was slight but not significant migration of the PKH26-labelled hMSCs from each group into the surrounding area, as determined by the lack of fluorescence signal away from the original central seeding area.

**Figure 6.**
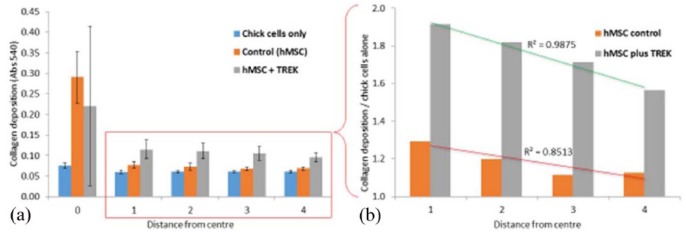
Collagen deposition by chick epiphyseal cells was enhanced by TREK1 nanoparticle–activated hMSCs. (a) Collagen production was increased by ~20% when chick epiphyseal cells were cultured with hMSCs. When hMSCs are mechanically stimulated by TREK1 magnetic nanoparticles, collagen production (predominantly from the surrounding chick cells) was increased by 60%–90%. (b) A closer examination of collagen deposition as a function of distance from the central hMSC colony shows that chick epiphyseal cells closer to the hMSCs produced more collagen (normalised against the chick-only control and presented as fold-change). Collagen production by chick cells was inversely proportional to their distance from the hMSCs, indicating a biochemical concentration gradient or direct cell–cell contact is required to stimulate upregulation of collagen synthesis. Error bars show standard deviation, *n* = 6; *R*^2^ values indicate coefficient of linear regression.

Collagen content of the cultures was determined by staining the wells with Sirius red dye (Figure 6). The CECs alone were shown to have a uniform distribution of collagen at each measurement point, whereas the addition of hMSCs resulted in a proportional increase in collagen deposition closest to the hMSCs (coefficient of linear regression, *R*^2^ = 0.8513). When stimulated with TREK1 nanoparticles, the collagen deposition across the entire plate was shown to be significantly enhanced (by 60%–90%) at each measurement point, and again was proportional to the proximity to the hMSC colony (coefficient of linear regression, *R*^2^ = 0.9875).

**Figure 7.**
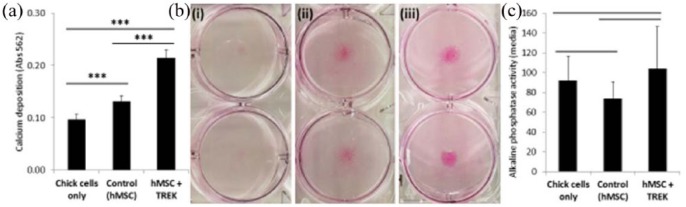
Mineralisation in the co-culture was significantly increased by the presence of hMSCs and activation of hMSC mechanotransduction. (a) After 28 days, calcium deposition was determined by staining the plates with alizarin red, which was then solubilised with cetylpyridinium chloride and quantified spectrometrically. Calcium deposition was enhanced by the presence of the hMSCs (37% increase in total calcium deposition) and further enhanced by the activation of hMSC mechanotransduction (128% increase in calcium deposition) compared to the controls lacking MSCs. Visual analysis of the mineral distribution (b) shows that the chick epiphyseal cells mineralise the extracellular matrix (i) and mineralisation in the groups containing MSCs was localised in the central region surrounding the hMSCs (ii) and TREK-activated MSCs (iii) – the central zone delimiting the MSCs is indicated by the blue circles. At the 28 day end point, there was no significant difference in alkaline phosphatase activity in the cell-culture media supernatant (c). Error bars show standard deviation, *n* = 6 and ***p < 0.001.

The co-cultures were subsequently analysed for changes in the extracellular matrix composition, starting with calcification/mineralisation across the well (Figure 7). All groups were cultured in semi-osteogenic media and in the presence of the magnetic array. Control cultures of CECs alone had mineralised extracellular matrix, but the amount of calcium deposition was increased 37% by the presence of hMSCs and further increased to 128% when the hMSCs were stimulated via TREK1 nanoparticles. Alizarin red staining showed the calcification to be centred on the area containing the hMSCs, but with the entire well showing evidence of calcification. At the 28 end point, there was no significant difference in alkaline phosphatase activity across any of the groups.

## Discussion

MSCs are being investigated as a powerful cell-based therapy, but their mechanism of action is still largely unknown.^15,16^ Reviews have generally reported that hMSC engraftment at implantation sites is variable with their long-term survival potentially restricted.^17^ Nevertheless, the ability of MSCs to effect tissue healing and repair in many instances is generally recognised, and so the hypothesis which emerges is that the stem cells may have a potent secretome that induces effective changes in the surrounding cells.^18,19^ Reports have also suggested that the secretome can just as commonly be catabolic rather than anabolic,^20^ so understanding and controlling the mechanisms that regulate the MSC secretome in vivo is beginning to attract attention as a method for controlling healing, intervening in disease progression and even diminishing the effects of ageing.^21^

Our previously published data from a chick foetal femur microinjected with human MSCs^4^ demonstrated osteogenic differentiation of the injected cells but also demonstrated effects remote from the cells in the surrounding tissues. These results lead us to the following two hypotheses: (1) hMSCs migrated through the developing cartilaginous tissue and themselves generated epiphyseal bone or (2) the cells did not significantly migrate, and instead, the increased osteogenesis resulted from hMSC–CEC signalling. To test these competing theories, we first measured the ability of the human MSCs to migrate across a transwell membrane towards the external magnet used to apply a torque to the magnetic nanoparticle on the cell membrane. While these cells did migrate more than control MSCs (without nanoparticles or magnet), their migration was much lower than chemotaxis towards FCS and not significantly different to experimental groups exposed to either the magnet or nanoparticles alone. Other groups have reported that chemotactic migration of MSCs in vitro towards gradients of IGF1 or platelet-derived growth factor (PDGF) are equal or greater to the positive FCS control used in our experiment.^22^ Our conclusions from this study are that at this dose of magnetic labelling, there are not sufficient magnetic forces to promote migration towards the magnet, thus this slightly enhanced migration in the TREK1-tagged hMSC in response to a magnetic field may be due to changes in focal adhesions, altered adherence and cytoskeletal remodelling caused by the downstream effects of mechanotransduction.^23^

To further investigate migration, we generated a simplified but robust co-culture assay, in which a droplet of TREK1 nanoparticle–tagged and fluorescently labelled hMSCs were placed in a small central region of a well plate and then surrounded by a confluent layer of chick cells. This 2D monolayer setup mimics the 3D organotypic model published in our earlier study^4^ yet allows closer interrogation of migration and extracellular matrix (ECM) formation without the difficulties of collagen autofluorescence which plague 3D cell tracking in organised tissues. In our co-culture experiment, hMSCs did not significantly migrate out from their central seeding area into the 2D regions covered with CECs with or without the TREK labelling and magnetic nanoparticles.

We found that the presence in the well of hMSCs increased total collagen production by ~20%, and when hMSCs are stimulated by TREK1Ab-conjugated magnetic nanoparticles, collagen production by surrounding chick cells is increased by 60%–90% over controls of just CECs alone. Collagen production by chick cells was inversely proportional to their distance from the hMSCs, indicating that a concentration gradient or direct cell–cell contact is required to stimulate upregulation of collagen synthesis. This finding is supported by multiple other published studies in which injected MSCs promoted collagen synthesis in vitro^24^ and in vivo^25^ especially when employed on cell types derived from joint tissues. The effects seen here and reported by Henstock et al.^4^ are generated from a relatively small number of hMSCs (10^3^ cells) acting on a much larger number of ‘host’ cells (10^6^ CECs in this case). This once again reflects the therapeutic potential of injected or implanted hMSCs to act as regulators and promoters of tissue regeneration in clinical applications such as bone non-union or osteoarthritis.^26^

We also observed that the presence of MSCs caused an increased incorporation of calcium into the extracellular matrix, and nanoparticle-stimulated MSCs promoted significantly increased mineralisation. Chick cells alone were capable of mineralising the matrix, while calcification was centred in regions containing MSCs and broadly distributed throughout the rest of the well – there did not appear to be the same proximity-based correlation as observed for collagen production. This reflects the nature of ECM mineralisation which relies on soluble factors in addition to ECM proteins such as osteocalcin, and the origin of the factors promoting mineralisation is unclear.^27^

It should be noted that increased collagen synthesis from surrounding cells is not necessarily synonymous with bone tissue regeneration, and indeed wound healing, scar formation and functional regeneration are a complex balance. When considered alongside the increase in mineralisation, the effects we observe appear to be osteoinductive, but clearly substantial further work is required to determine the quality of the ‘bone’ formed under these circumstances and the types of collagens produced. A major outstanding question from this work is the relative contribution of critical matrix proteins such as osteocalcin and the larger family of collagen isoforms to the mineralising matrix produced by the CECs. Our further investigations in the area focus on the ability of cells to generate a functional bone repair through mechanisms which originate with mechanoactivation of signalling pathways.^2^

Our previously published research has demonstrated that mechanotransduction pathways can be activated by magnetic nanoparticles conjugated with antibodies and ligands which bind mechanoreceptors – for example, integrins and ion channels, and we have translated this from in vitro research through to a large animal model of bone repair.^2^ Mechanical stimulation is a strong promoter of stem-cell differentiation and tissue growth, generating tissues which are mechanically adapted and have functional extracellular matrix.^28^ The MICA technique may therefore present a useful, injectable therapeutic in which host cells are tagged as a minimally invasive procedure and mechanically activated. The injected cells can then go forward to regenerate tissue and furthermore influence surrounding tissue regeneration through paracrine signalling to endogenous cell communities.^29,30^

Our conclusions from this study are that the hMSC secretome (in an osteogenic media environment) is generally osteoinductive, resulting in enhancement of bone formation from surrounding tissue. Furthermore, mechanical stimulation via mechanosensitive stretch-activated ion channels (in this case TREK1) may increase the osteogenic properties of the secretome. In this study, we did not conduct any detailed investigation into the contents of the secretome, but this will form a significant part of our future research. The composition of the MSC secretome is currently under investigation by a number of research groups, since it has clear applications as a cell-free driver of biology with uses throughout translational and regenerative medicine.^31,32^ Our subsequent plans are to quantify the conventional signalling molecules (e.g., prostaglandins^33^ and cytokines) and growth factors secreted by the hMSCs but recognise that the secretome may equally contain a myriad of regulatory molecules including microRNAs.^34^ This is clearly both an interesting and challenging body of work, but the potential for using magnetic nanoparticles to remotely control anabolic cell signalling could be transformative.
